# Hypointense signals in the infrapatellar fat pad assessed by magnetic resonance imaging are associated with knee symptoms and structure in older adults: a cohort study

**DOI:** 10.1186/s13075-016-1130-y

**Published:** 2016-10-12

**Authors:** Weiyu Han, Dawn Aitken, Zhaohua Zhu, Andrew Halliday, Xia Wang, Benny Antony, Flavia Cicuttini, Graeme Jones, Changhai Ding

**Affiliations:** 1 Menzies Institute for Medical Research, University of Tasmania, Private Bag 23, Hobart, TAS 7000 Australia; 2 Department of Orthopedics, 3rd Affiliated Hospital of Southern Medical University, Guangzhou, China; 3 Arthritis Research Institute, 1st Affiliated Hospital, Anhui Medical University, Hefei, Anhui China; 4 Department of Radiology, Royal Hobart Hospital, Hobart, TAS Australia; 5 Department of Epidemiology and Preventive Medicine, Monash University, Melbourne, VIC Australia

**Keywords:** Infrapatellar fat pad, Osteoarthritis, Signal intensity, Cartilage defects, Bone marrow lesions, Pain

## Abstract

**Background:**

There are few clinical and epidemiological studies reporting the association between abnormal changes within the IPFP and knee osteoarthritic changes. This study aims to describe the associations between hypointense signals in the infrapatellar fat pad (IPFP) and knee structural change and symptoms in older adults.

**Methods:**

Participants (*n* = 874) were selected randomly from local community and followed up 2.7 years later (range 2.6–3.3 years). T1- or T2-weighted fat-suppressed magnetic resonance imaging (MRI) was assessed for IPFP hypointense signal, cartilage volume, cartilage defects, and bone marrow lesions (BMLs). Knee pain was assessed by self-administered Western Ontario and McMaster Osteoarthritis Index (WOMAC) questionnaire. Radiographic osteoarthritis was assessed using the OARSI atlas.

**Results:**

Cross-sectionally, hypointense signals in the IPFP were significantly associated with a higher risk of knee cartilage defects at all sites, tibiofemoral BMLs and knee pain in multivariable analyses. Longitudinally, baseline signal abnormalities were significantly and positively associated with increases in knee cartilage defects (OR: 2.27, 95 % CI: 1.61–3.21), BMLs (OR: 1.91, 95 % CI: 1.39–2.62), and knee pain (OR: 1.36, 95 % CI: 1.05–1.76) in multivariable analyses. The associations with cartilage defects remained significant after adjustment for BMLs, but the associations with BMLs and knee pain decreased in magnitude or became non-significant after further adjustment for cartilage defects.

**Conclusions:**

Hypointense signals in the IPFP were associated primarily with increased knee cartilage defects and also with BMLs and knee symptoms in cross-sectional and longitudinal analyses, suggesting the abnormality represented by this signal has a potentially important role in osteoarthritis progression.

**Electronic supplementary material:**

The online version of this article (doi:10.1186/s13075-016-1130-y) contains supplementary material, which is available to authorized users.

## Background

Osteoarthritis (OA) is the most prevalent chronic joint disorder, characterized by pain and progressive deterioration of joint structures, and is strongly associated with risk factors such as age, female sex and obesity [1]. The most commonly affected joint is the knee, and the whole knee joint structures including articular cartilage, subchondral bone, synovium, ligaments, meniscus and periarticular fat pad can be affected in the course of knee OA [2]. Imaging biomarkers, especially from magnetic resonance imaging (MRI), have been used in OA research for over a decade [3]. Because of its advantage in direct visualization of morphology and integrity of the whole joint, MRI has been considered as a sensitive and accurate tool to assess cartilage loss, subchondral bone abnormalities, synovitis, and ligament and meniscal lesions [4–6]. Quantitative or semiquantitative scoring systems have been developed for evaluating these structural changes in OA [4–6].

The infrapatellar fat pad (IPFP), the local fat around the knee joint, may play an important role in the initiation and progression of knee OA [7, 8]. Biomechanically, it can promote efficient lubrication, reduce impact loading and absorb forces generated through the knee joint, which may be protective against OA [9]. Biochemically, it can produce various pro-inflammatory cytokines and adipokines, which may be deleterious to the knee joint [10–13]. Pathological examination of IPFP obtained from patients with end-stage OA found that vascular neoformations, fibrosis, and chronic inflammation were present in these specimens [14]. Dragoo et al. have suggested that sagittal MRI can be used to assess abnormal IPFP quality, including fibrosis, inflammation, oedema and mass-like lesions [15].

So far, there are few clinical and epidemiological studies reporting the association between abnormal changes within the IPFP and knee osteoarthritic changes. Higher signal intensity change around the IPFP assessed by T2-weighted MRI has been considered as a surrogate for peripatellar synovitis [16, 17]. Our previous study reported that high signal intensity alteration within the IPFP was associated with knee symptoms and structural changes in older adults [18]. While hyperintense signals within the IPFP on T2-weighted MRI can indicate inflammation, acute haemorrhage and/or oedema, hypointense signals within the IPFP on T2-weighted MRI may indicate fibrosis [15]. So far, there are no studies reporting whether hypointense signals within the IPFP are associated with symptoms and structures in knee OA. The aim of this study was to describe whether hypointense signals within the IPFP measured by T2-weighted MRI are associated with symptoms or joint structural abnormalities cross-sectionally and longitudinally in older adults.

## Methods

### Subjects

This study was conducted as part of the Tasmanian Older Adult Cohort (TASOAC) study, an ongoing prospective, population-based study that was aimed at identifying the environmental, genetic, and biochemical factors associated with the development and progression of OA. Men and women aged 50–80 years in 2002 were selected from the electoral roll in southern Tasmania (population, 229,000) using sex-stratified simple random sampling without replacement (response rate, 57 %). Baseline measures were conducted from April 2002 to September 2004, and the follow-up was conducted from September 2004 to February 2007 (mean 2.7 years, range 2.6–3.3 years). Institutionalized persons and subjects with contraindications to MRI and diagnosed rheumatoid arthritis were excluded. This study consisted of 874 participants who had knee MRI scans at baseline.

### Anthropometrics

Height was measured to the nearest 0.1 cm (with shoes, socks, and headgear removed) using a stadiometer. Weight was measured to the nearest 0.1 kg (with shoes, socks, and bulky clothing removed) by using a single pair of electronic scales (Delta Model 707, Seca, Hamburg, Germany) that were calibrated using a known weight at the beginning of each clinic. Body mass index [BMI, weight (kg)/height (m^2^)] was also calculated.

### WOMAC pain assessment

The assessment of knee pain (when walking on flat surface, when going up/down stairs, at night while in bed, when sitting/lying and when standing) was self-administered, using the Western Ontario and McMaster Osteoarthritis Index (WOMAC) with a 10-point scale from 0 (no pain) to 9 (most severe) [19]. A total score for knee pain (0 to 45) was determined by each component score, and the presence of knee pain was defined as a total score or a subscale score of ≥ 1. An increase in knee pain was defined as a change in the score of ≥ 1.

### Knee radiographic assessment

All subjects performed a standing anteroposterior semiflexed view of the right knee with 15 degrees of fixed knee flexion, and radiographs were individually assessed for joint space narrowing (JSN) and osteophytes on a scale of 0–3 (0 = normal and 3 = most severe) by using the Osteoarthritis Research Society International (OARSI) atlas developed by Altman et al. [20]. The osteophytes and JSN scores were summed as the knee total radiographic OA (ROA) score, of which 1 or greater was used to define the presence of knee ROA, as previously described [21].

### Magnetic resonance imaging assessment

MRI scans of the right knees were performed at baseline and follow-up. Knees were imaged in the sagittal plane on a 1.5-T whole body magnetic resonance unit (Picker, Cleveland, OH, USA) with the use of a commercial transmit-receive extremity coil. The following image sequences were used: (1) a T1-weighted fat saturation three-dimensional gradient recall acquisition in the steady state; flip angle 30 degrees; repetition time 31 ms; echo time 6.71 mc; field of view 16 cm; 60 partitions; 512 × 512 matrix; acquisition time 11 min 56 sec; one acquisition. Sagittal images were obtained at a partition thickness of 1.5 mm and an in-plane resolution of 0.31 × 0.31 (512 × 512 pixels); (2) a T2-weighted fat saturation two-dimensional fast spin echo, flip angle 90°, repetition time 3067 ms, echo time 112 ms, field of view 16 cm, 15 partitions, 256 × 256-pixel matrix; sagittal images were obtained at a slice thickness of 4 mm with a interslice gap of 1.0 mm.

**Fig. 1:**
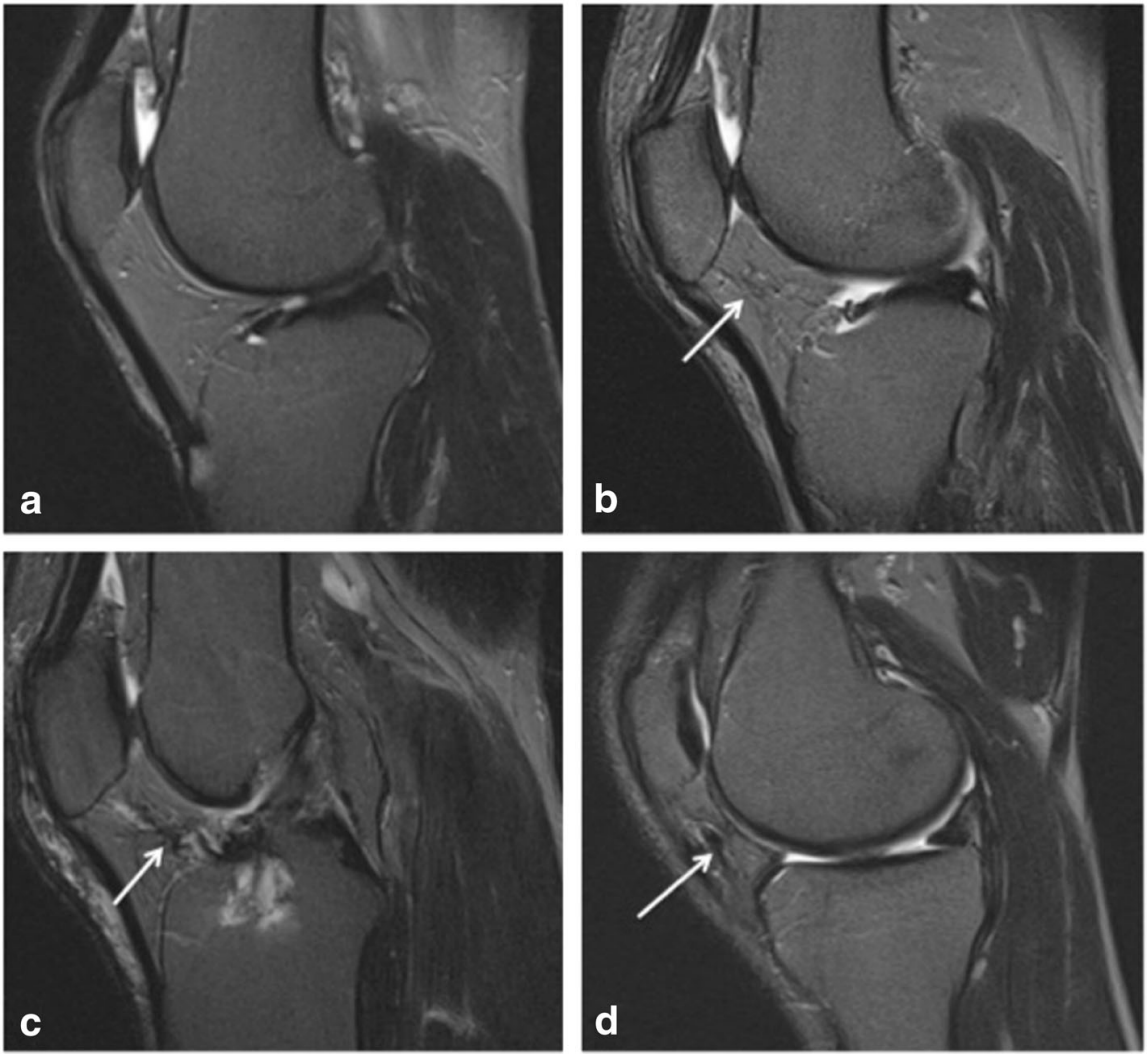
Hypointense signals on sagittal T2-weighted images with fat saturation. **a** Normal IPFP; **b** grade 1 hypointense signals of IPFP (*arrow*); **c** grade 2 hypointense signals of IPFP (*arrow*); **d** grade 3 hypointense signals of IPFP (*arrow*)

Hypointense signals within the IPFP were scored by counting imaging slices with this abnormality: grade 0 = none; grade 1 = 1–2 slices, grade 2 = 3–5 slices, grade 3 = ≥6 slices. This measurement was conducted by two experienced orthopaedists (HW and ZZ) trained by an experienced radiologist (HA), and determined using T2-weighted MR images (Fig. 1). Intraobserver and interobsever reliabilities were assessed in 100 subjects with an intraclass correlation coefficient (ICC) of 0.94 and an interobserver correlation coefficient of 0.88.

Knee cartilage volume was assessed on T1-weighted MR images with image processing on an independent workstation, as previously described [22–24]. The total cartilage volume was divided into patellar, medial and lateral tibial cartilage volume by manually drawing disarticulation contours around the cartilage boundaries, section by section, which were then resampled for the final three-dimensional rendering [22, 23]. The coefficients of variation (CVs) for this method in our hands were 2.1–2.6 % [22, 23]. Changes in cartilage volume were calculated as: change per annum = (follow-up volume – baseline volume)/time between two scans in years.

Cartilage defects (0–4 scale) were determined at the medial tibial, medial femoral, lateral tibial, lateral femoral, and patellar sites as previously described [25, 26] as follows: grade 0 = normal cartilage; grade 1 = focal blistering and intracartilaginous low-signal intensity area with an intact surface; grade 2 = irregularities on the surface or bottom and loss of thickness < 50 %; grade 3 = deep ulceration with loss of thickness > 50 %; grade 4 = full-thickness chondral wear with exposure of subchondral bone. The presence of cartilage defects was defined as a cartilage defect score of ≥ 2 at any site. Intraobserver reliabilities were 0.89–0.94 and interobserver reliabilities were 0.85–0.93 [25]. An increase in cartilage defects was defined as a change in cartilage defects of ≥ 1.

Subchondral bone marrow lesions (BMLs) were defined as discrete areas of increased signal adjacent to the subcortical bone at the medial and lateral tibia and femur on T2-weighted MR images using a semi-quantitative (0–3) scoring system. The intraobserver reliability ranged between 0.89–1.00, as previously described [27]. An increase in BMLs was defined as a change in BMLs of ≥ 1.

Tibial plateau bone area was determined by manually measuring on axial T1-weighted MR images, as previously described [21].

### Data analysis

Student *t* or χ^2^ tests were used to compare means or proportions, respectively. Multivariable linear regression analyses were used to examine the associations between IPFP hypointense signals (independent variable) and knee cartilage volume or change in cartilage volume (dependent variables) after adjustment for age, sex, BMI, ROA, tibial bone area, and/or baseline cartilage volume (for change in cartilage volume) with further adjustment for cartilage defects or BMLs. Multivariable binary logistic regression analyses were used to examine the associations between IPFP hypointense signals (independent variable) and presences of knee joint space narrowing, osteophytes, as well as baseline or increases in knee cartilage defects, BMLs and WOMAC measures (dependent variables), after adjustment for covariates.

A *p* value < 0.05 (two-tailed) or a 95 % confidence interval (CI) not including the null point (for linear regression) or 1 (for logistic regression) was considered as statistical significance. All statistical analyses were performed on IBM SPSS version 20.0 for Windows (IBM Corp., Armonk, NY, USA).

## Results

A total of 874 subjects between 50 and 80 years of age (mean, 62.1 years) took part in the present study. There were no significant differences in demographic factors (age, sex, and BMI) between these participants and those excluded (n = 226) (data not shown). Over 2.6 years, 104 subjects were lost to follow-up study due to: 25 deceased, 18 moved to other states or overseas, 12 had joint replacement, 24 physically unable, and 25 no reason specified. The remaining 770 subjects completed the follow-up study and the first 357 had the second MRI scans but not the others as the MRI machine in the hospital was decommissioned. There were no significant differences between these subjects and those without follow-up MRI, as previously described [28].

Table 1 describes characteristics of the study population. There was no significant difference in age, patellar cartilage volume and knee pain between subjects with and without IPFP hypointense signals; but the group with IPFP hypointense signals had a greater proportion of men, and higher prevalence of JSN, osteophytes, BMLs, cartilage defects, as well as higher BMI. Additionally, these subjects had greater tibial cartilage volume and tibial bone area.

**Table 1:** Baseline characteristics of participants split by presence of IPFP hypointense signal

	IPFP hypointense signal	IPFP hypointense signal	*p* value
	No (*N* = 305)	YES (*N* = 569)	
Age (year)	61.6 (6.9)	62.4 (7.5)	0.090
Female sex (%)	**65.9**	**41.7**	**<0.001**
Body mass index (kg/m^2^)	**27.0 (4.5)**	**28.1 (4.7)**	**<0.001**
Medial tibial cartilage volume (ml)	**2.2 (0.6)**	**2.4 (0.6)**	**<0.001**
Lateral tibial cartilage volume (ml)	**2.6 (0.7)**	**2.8 (0.7)**	**<0.001**
Patella cartilage volume (ml)	3.2 (0.9)	3.2 (1.0)	0.235
Medial tibial bone area (cm^2^)	**19.9 (2.9)**	**21.4 (3.0)**	**<0.001**
Lateral tibial bone area (cm^2^)	**11.4 (1.9)**	**12.5 (2.2)**	**<0.001**
Medial joint space narrowing (%)	**32.9**	**63.3**	**<0.001**
Lateral joint space narrowing (%)	**15.2**	**28.2**	**<0.001**
MTF osteophytes (%)	**1.8**	**10.0**	**<0.001**
LTF osteophytes (%)	**0.7**	**5.5**	**0.001**
BML present (%)	**29.2**	**40.1**	**0.001**
MTF cartilage defects (%)	**10.2**	**30.8**	**<0.001**
LTF cartilage defects (%)	**11.3**	**26.7**	**<0.001**
Patellar cartilage defects (%)	**29.4**	**44.3**	**<0.001**
Knee pain (%)	46.2	51.4	0.144

Two-tailed *t* tests were used for differences between means, and χ2 tests were used for proportions (percentages). Significant differences are shown in bold. Mean (SD) except for percentages

*IPFP* infrapatellar fat pat, *MTF* medial tibiofemoral, *LTF* lateral tibiofemoral, *BML* bone marrow lesions

**Fig. 2:**
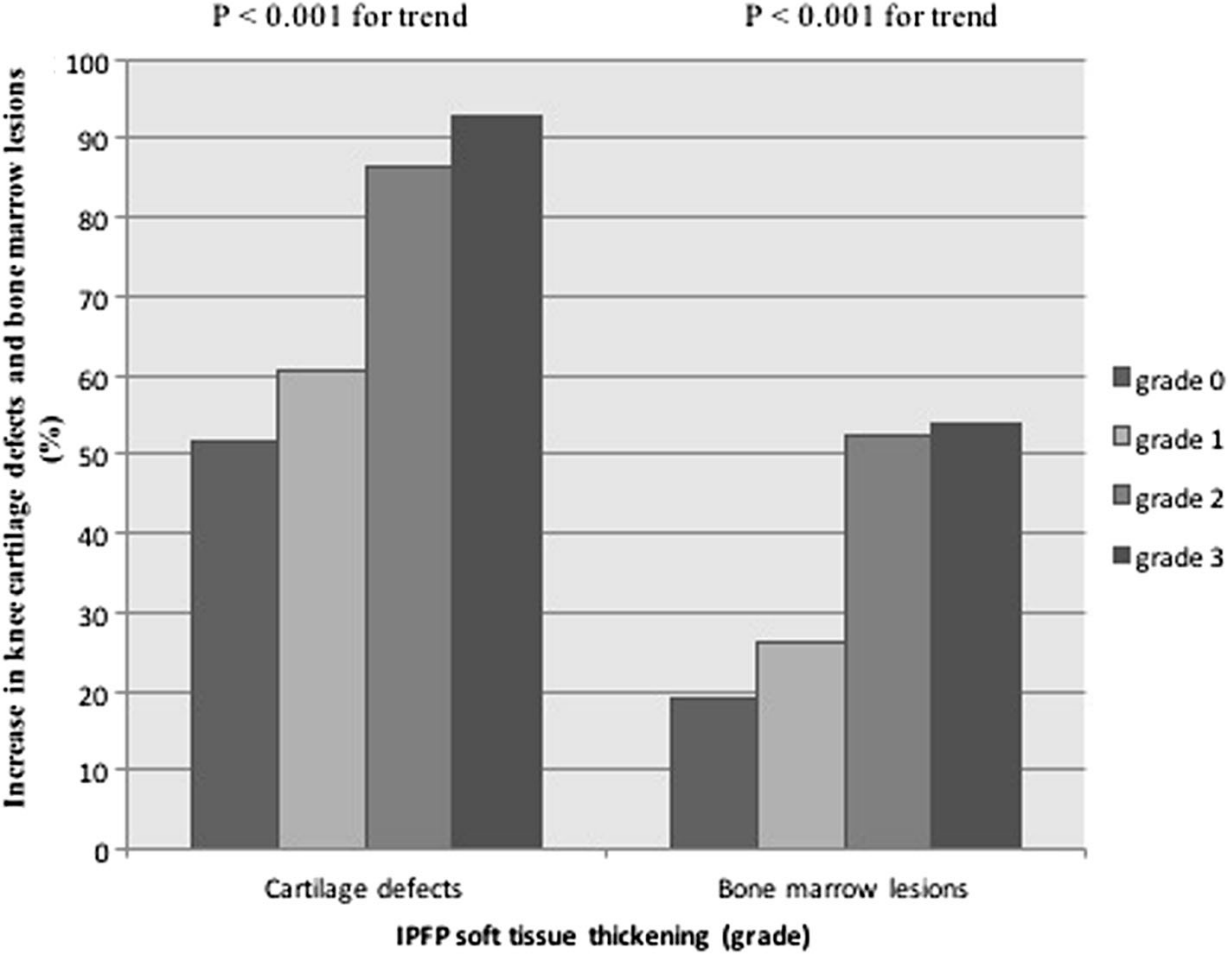
Association of IPFP hypointense signals with increase in knee cartilage defects and bone marrow lesions. *IPFP* infrapatellar fat pad

IPFP hypointense signals were significantly and positively associated with baseline (data not shown) and increases in (Fig. 2) cartilage defects at all compartments in unadjusted analyses. They were significantly and positively associated with all cartilage defects after adjustment for age, sex, BMI, and radiographic OA cross-sectionally and longitudinally (Table 2). These associations remained significant after further adjustment for BMLs, except that the longitudinal association at the patellar site decreased in magnitude and became of borderline significance (Table 2).

**Table 2:** Associations of IPFP hypointense signals with baseline knee cartilage defects and increases in knee cartilage defects over 2.6 years

	Multivariable^a^	Multivariable^b^
	OR (95 % CI)	OR (95 % CI)
*Baseline cartilage defects*		
Any cartilage defects	2.38 (1.87, 3.03)	2.24 (1.74, 2.87)
Medial tibiofemoral	2.93 (2.24, 3.83)	2.70 (2.05, 3.54)
Lateral tibiofemoral	2.62 (2.02, 3.42)	2.57 (1.95, 3.38)
Patellar	1.84 (1.47, 2.31)	1.93 (1.50, 2.47)
*Increase in knee cartilage defects*		
Any cartilage defects	2.27 (1.61, 3.21)	2.08 (1.46, 2.97)
Medial tibiofemoral	1.62 (1.21, 2.19)	1.55 (1.15, 2.09)
Lateral tibiofemoral	1.46 (1.09, 1.96)	1.39 (1.03, 1.88)
Patellar	1.38 (1.00, 1.90)	1.36 (0.99, 1.88)

Dependent variables: baseline cartilage defects or increases in knee cartilage defects (yes v no); independent variables: IPFP hypointense signals (per grade)

*IPFP* infrapatellar fat pat

^a^Adjusted for age, sex, BMI, and radiographic osteoarthritis

^b^Further adjustment for bone marrow lesions

Cross-sectionally, IPFP hypointense signals were not significantly associated with medial and lateral tibial cartilage volume, but significantly and negatively associated with patellar cartilage volume after adjustment for age, sex, BMI, radiographic OA and tibial bone area (Additional file 1: Table S1). This significant association disappeared after further adjustment for patellar cartilage defects but remained unchanged after further adjustment for patellar BMLs (Additional file 1: Table S1). Longitudinally, IPFP hypointense signals were negatively and significantly associated with change in lateral tibial cartilage volume, but not with changes in medial tibial and patellar cartilage volume, after adjustment for age, sex, BMI, radiographic OA, tibial bone area and baseline cartilage volume, and this association remained after further adjustment for cartilage defects or BMLs (Additional file 1: Table S1).

In cross-sectional analyses, IPFP hypointense signals were significantly and positively associated with any BMLs and tibiofemoral BMLs after adjustment for age, sex, BMI and radiographic OA. The associations decreased in magnitude and became non-significant after further adjustment for cartilage defects (Table 3). Longitudinally, IPFP hypointense signals were significantly and positively associated with increases in BMLs at all sites before (Fig. 2) and after adjustment for age, sex, BMI and radiographic OA (Table 3). After further adjustment for cartilage defects, the associations decreased in magnitude and became non-significant at lateral tibiofemoral and patellar sites (Table 3).

**Table 3:** Associations between IPFP hypointense signals and baseline bone marrow lesions and increases in bone marrow lesions over 2.6 years

	Multivariable^a^	Multivariable^b^
	OR (95 % CI)	OR (95 % CI)
*Baseline bone marrow lesions*		
Any bone marrow lesions	1.64 (1.32, 2.03)	1.11 (0.87, 1.42)
Medial tibiofemoral	1.77 (1.39, 2.25)	1.24 (0.95, 1.63)
Lateral tibiofemoral	1.40 (1.08, 1.80)	1.03 (0.78, 1.36)
Patellar	1.21 (0.93, 1.57)	0.83 (0.61, 1.13)
*Increases in bone marrow lesions*		
Any bone marrow lesions	1.91 (1.39, 2.62)	1.45 (1.02, 2.04)
Medial tibiofemoral	2.11 (1.46, 3.07)	1.59 (1.06, 2.39)
Lateral tibiofemoral	1.66 (1.17, 2.35)	1.36 (0.93, 1.97)
Patellar	1.50 (1.04, 2.15)	1.34 (0.92, 1.95)

Dependent variables: baseline bone marrow lesions or increases in bone marrow lesions (yes v no); independent variables: IPFP hypointense signals (per grade)

*IPFP* infrapatellar fat pat

^a^Adjusted for age, sex, BMI, and radiographic osteoarthritis

^b^Further adjustment for cartilage defects

IPFP hypointense signals were significantly and positively associated with total knee pain, pain when going up/down stairs, at night while in bed, and when sitting/lying after adjustment for age, sex, BMI, and radiographic OA in cross-sectional analyses, but these significant associations disappeared after further adjustment for cartilage defects or BMLs except for pain when at night while in bed (Table 4). Longitudinally, IPFP hypointense signals were significantly associated with an increase in total knee pain, pain when walking on a flat surface, pain when going up/down stairs and when standing (Table 4), but these became non-significant after further adjusting for cartilage defects. The associations between IPFP hypointense signals and an increase in knee pain decreased in magnitude but became non-significant for all knee pain subscales (except pain when going up/down stairs) after further adjustment for BMLs (Table 4).

**Table 4:** Association of IPFP hypointense signals with WOMAC measures and increases in WOMAC measures over 2.6 years

	Multivariable^a^	Multivariable^b^	Multivariable^c^
	OR (95 % CI)	OR (95 % CI)	OR (95 % CI)
*Baseline WOMAC measures*			
Total knee pain	1.27 (1.03, 1.56)	1.07 (0.85, 1.35)	1.15 (0.93, 1.43)
Pain on flat surface	1.22 (0.97, 1.55)	1.02 (0.79, 1.33)	1.13 (0.88, 1.44)
Pain on stairs	1.26 (1.02, 1.55)	1.05 (0.84, 1.32)	1.14 (0.92, 1.42)
Pain in bed	1.47 (1.16, 1.85)	1.42 (1.10, 1.82)	1.42 (1.12, 1.79)
Pain when sitting	1.29 (1.02, 1.64)	1.25 (0.96, 1.62)	1.24 (0.97, 1.58)
Pain when standing	1.22 (0.96, 1.55)	1.10 (0.85, 1.43)	1.12 (0.87, 1.43)
*Increases in WOMAC measures*			
Total knee pain	1.36 (1.05, 1.76)	1.32 (0.99, 1.74)	1.30 (1.00, 1.69)
Pain on flat surface	1.52 (1.08, 2.14)	1.22 (0.83, 1.79)	1.33 (0.94, 1.89)
Pain on stairs	1.51 (1.14, 2.01)	1.34 (0.98, 1.82)	1.42 (1.06, 1.89)
Pain in bed	1.18 (0.85, 1.63)	1.04 (0.73, 1.49)	1.14 (0.82, 1.58)
Pain when sitting	1.25 (0.88, 1.78)	1.14 (0.77, 1.67)	1.20 (0.84, 1.71)
Pain when standing	1.44 (1.02, 2.03)	1.22 (0.84, 1.78)	1.38 (0.98, 1.97)

Dependent variables: baseline WOMAC measures or increases in WOMAC measures (yes vs. no); independent variables: IPFP hypointense signals (per grade)

*IPFP* infrapatellar fat pat, *WOMAC* Western Ontario and McMasters osteoarthritis index

^a^Adjusted for age, sex, BMI, and radiographic osteoarthritis

^b^Further adjustment for cartilage defects

^c^Further adjustment for bone marrow lesions but not for cartilage defects

In cross-sectional analyses, IPFP hypointense signals were significantly and positively associated with ROA (OR: 2.91, *p* < 0.001), tibiofemoral joint space narrowing (OR: 2.72 and 1.59, respectively, for medial and lateral compartments; both *p* < 0.01), and tibiofemoral osteophytes (OR: 4.25 and 3.34, respectively, for medial and lateral compartments; both *p* < 0.001), after adjustment for age, sex and BMI.

Higher grade of IPFP hypointense signals was significantly associated with smaller IPFP maximal area after adjustment for age, gender, BMI, and total tibial bone area (Additional file 2: Figure S1). The associations of IPFP hypointense signals with the above outcome measures remained unchanged after further adjustment for IPFP maximal area (data not shown).

## Discussion

This study is the first study to investigate the association of IPFP hypointense signals with knee structural and symptom changes in older adults. We found that IPFP hypointense signals were cross-sectionally associated with increased knee symptoms, cartilage defects, BMLs, and radiographic OA, and with reduced patellar cartilage volume and IPFP maximal area. Longitudinally, these signal intensity changes predicted increases in cartilage defects at all sites and BMLs, loss of lateral tibial cartilage volume and increases in knee symptoms. The associations with knee cartilage defects remained significant after adjustment for BMLs, but the associations with BMLs and knee pain were weakened after adjustment for cartilage defects. This suggests that IPFP hypointense signals are associated with increased knee cartilage defects primarily and with knee BMLs and pain secondarily in older adults.

IPFP, an intracapsular but extrasynovial structure [29], is situated in the knee under the patella, between the patellar tendon, femoral condyle and tibial plateau [7], and is structurally similar to subcutaneous adipose tissue [30]. As a deformable soft tissue within the anterior compartment of the knee joint, it can adapt to change contours of the articular surface and is able to distribute synovial fluid within the joint cavity to reduce the articular surface cushion, besides supporting the feasibility of intra-articular ligaments [9]. Composed of a fibrous scaffold with adipose tissue and synovial recesses within it, the IPFP can secrete cytokines, adipokines, and lipid mediators [10–13, 31]. Therefore, the IPFP may play a biphasic role in the pathologic progression of knee abnormalities.

MRI has been used to assess signal alterations within or around the IPFP, and the high signal intensity alteration was mainly considered as synovial inflammation or Hoffa synovitis [16, 32]. In addition, hypointense signals closing to the synovium within or around the IPFP was regarding as chronic synovitis [33, 34]. Hoffa synovitis is recognised as a key imaging biomarker for knee OA [6], and can predict the progression of knee OA [32]. Our previous study also suggested that high signal intensity alteration within the IPFP was associated with knee structural and symptomatic changes in older adults [18]. As IPFP signal intensity alteration may reflect different pathological changes, more studies are required to assess the roles of IPFP signal intensity changes in knee OA.

So far, there have been no studies reporting the clinical significance of IPFP hypointense signals observed on T2-weighted fat-saturated MRI images. Low signal intensity changes within the IPFP may represent fibrosis or postoperative scarring [29, 35], chronic inflammation progressing from acute inflammation of the synovium due to microtrauma of this tissue [36], or synovial thickening or fibrosis [33, 37]. A study compared low signal intensity changes in plantar fat pad on MRI with histological changes and reported that these signal changes corresponded to fibrosis [38]. This fibrosis can be induced by chronic inflammation in the synovium [36], or periarticular surgeries or trauma around knees [29]. Although the roles of synovitis, surgical history and trauma in knee OA have been identified [39, 40], there has been no evidence showing that low signal intensity within IPFP assessed by MRI is associated with symptoms and knee structural changes in the knee.

In this study, we found that in older adults, IPFP hypointense signals were consistently associated with knee cartilage defects cross-sectionally and longitudinally, independent of factors such as BMLs. They were also associated with BMLs and reduced cartilage volume cross-sectionally and longitudinally, but these associations were largely dependent of cartilage defects. Further, there was significant association between IPFP hypointense signals and knee pain, but again, these associations were largely dependent of cartilage defects rather than BMLs. These suggest that IPFP hypointense signals may induce knee structural changes and symptoms starting from cartilage defects.

The mechanisms underlying the associations between IPFP low signal intensity and knee OA measures are largely unknown. These hypointense signals within the synovial membrane have been considered as synovial fibrosis and were corresponding to chronic synovitis [33, 34]. This chronic synovitis can contribute to cartilage degradations and knee pain [41]. Other pathological changes such as adipocyte necrosis or adipose fibrosis may be observed as hypointense signals within the IPFP in MR imagines. Fibrosis is an abnormal tissue healing process that occurs sequentially from an inflammatory response to surgery or injury of the knee and severe fibrosis was found in 33 % of IPFP biopsy specimens resected from patients with end-stage knee OA [14]. Fibrosis was also found in monoiodoacetate-induced OA models and was associated with knee pain [36]. In addition, a study focusing on the effect of strenuous running on IPFP histological changes in a rat OA model reported that fibrosis within IPFP was associated with excess physical activities and related to knee pain [42]. Fibrosis within IPFP may increase cartilage contact pressures and decrease the ability of absorbing the shock through the knee, and thus induce the degradation of neighbouring knee structures including cartilage and subchondral bone. Furthermore, our current study found that this abnormal signal was negatively associated with IPFP maximum area, suggesting it may decrease this absorbing ability through reducing the IPFP size.

The main strength of this cohort study lies in a large sample size with the comprehensive MRI structural measurements. There are several potential limitations. First, the response rate at baseline was 57 %, possibly due to the extensive protocol, which did leave the possibility open for selection bias. However, there were no significant differences in age, gender and BMI between those who responded and those did not. We also had high rates of retention (82 %) to offset this. Second, we did not have radiographic OA measurements at the follow-up because X-ray is insensitive for change over this short period, so we are unable to determine the association between IPFP quality and change in radiographic OA. Third, measurement error may influence results. However, all measures were highly reproducible suggesting this is unlikely. Lastly, histological examinations were not able to be performed in this community-based study, so the pathological changes associated with IPFP low signal intensity are unknown.

## Conclusions

In conclusion, hypointense signals in the IPFP were associated primarily with increased knee cartilage defects and also with BMLs and knee symptoms in cross-sectional and longitudinal analyses, suggesting the abnormality represented by this signal has a potentially important role in osteoarthritis progression.

## Additional files

Additional file 1: Table S1. Associations of IPFP hypointense signals with baseline knee cartilage volume and change in knee cartilage volume over 2.6 years. (DOC 31 kb)
Additional file 2: Figure S1. Association between IPFP maximum area and hypointense signals. *IPFP* infrapatellar fat pad. (PNG 59 kb)

## Abbreviations

BMI: body mass index
BMLs: bone marrow lesions
CI: confidence interval
CVs: coefficients of variation
ICC: intraclass correlation coefficient
IPFP: infrapatellar fat pad
JSN: joint space narrowing
MRI: magnetic resonance imaging
OA: osteoarthritis
OARSI: Osteoarthritis Research Society International
ROA: radiographic osteoarthritis
TASOAC: Tasmanian Older Adult Cohort
WOMAC: Western Ontario and McMaster Osteoarthritis
