# Supplementary material for: Hypointense signals in the infrapatellar fat pad assessed by magnetic resonance imaging are associated with knee symptoms and structure in older adults: a cohort study
Source: Arthritis Res Ther. 2016 Oct 12;18:234. doi: 10.1186/s13075-016-1130-y (PMC5059934; doi:10.1186/s13075-016-1130-y)
Supplement: Additional file 1: Table S1. — Associations of IPFP hypointense signals with baseline knee cartilage volume and change in knee cartilage volume over 2.6 years. (DOC 31 kb) [file 13075_2016_1130_MOESM1_ESM.doc]

**Table S1.** Associations of IPFP hypointense signals with baseline knee cartilage volume and change in knee cartilage volume over 2.6 years

|  | Multivariable*  β (95% CI) | Multivariable** β (95% CI) | Multivariable***  β (95% CI) |
| --- | --- | --- | --- |
| *Baseline cartilage volume*  Medial tibial  Lateral tibial  Patellar  *Change in cartilage volume*  Medial tibial  Lateral tibial  Patellar | -20.2 (-66.4, 26.1)  -8.7 (-64.3, 47.0)  **-165.6 (-244.4, -86.7)**  -6.3 (-23.2, 10.6)  **-19.7 (-35.6, -3.8)**  -15.2 (-39.6, 9.1) | 17.5 (-30.2, 65.2)  46.7 (-7.9, 101.2)  -47.0 (-112.9, 18.9)  -3.6 (-21.1, 13.9)  **-17.0 (-33.3, -0.7)**  -5.7 (-29.4, 17.9) | -10.3 (-57.0, 36.4)  -5.4 (-61.2, 50.3)  **-154.9 (-232.7, -77.2)**  -5.0 (-22.1, 12.0)  **- 17.8 (-33.6, -2.0)**  -12.5 (-36.5, 11.5) |

Dependent variables: knee cartilage volume or change in knee cartilage volume per annum (mm^3^); independent variables: IPFP hypointense signals (per grade). *Adjusted for age, sex, BMI, radiographic osteoarthritis, tibial bone area, and/or baseline cartilage volume (for change in cartilage volume). **Further adjustment for cartilage defects. *** Further adjustment for bone marrow lesions but not for cartilage defects. IPFP: infrapatellar fat pat
