# Supplementary figures and images for: Hypointense signals in the infrapatellar fat pad assessed by magnetic resonance imaging are associated with knee symptoms and structure in older adults: a cohort study
Source: Arthritis Res Ther. 2016 Oct 12;18:234. doi: 10.1186/s13075-016-1130-y (PMC5059934; doi:10.1186/s13075-016-1130-y)

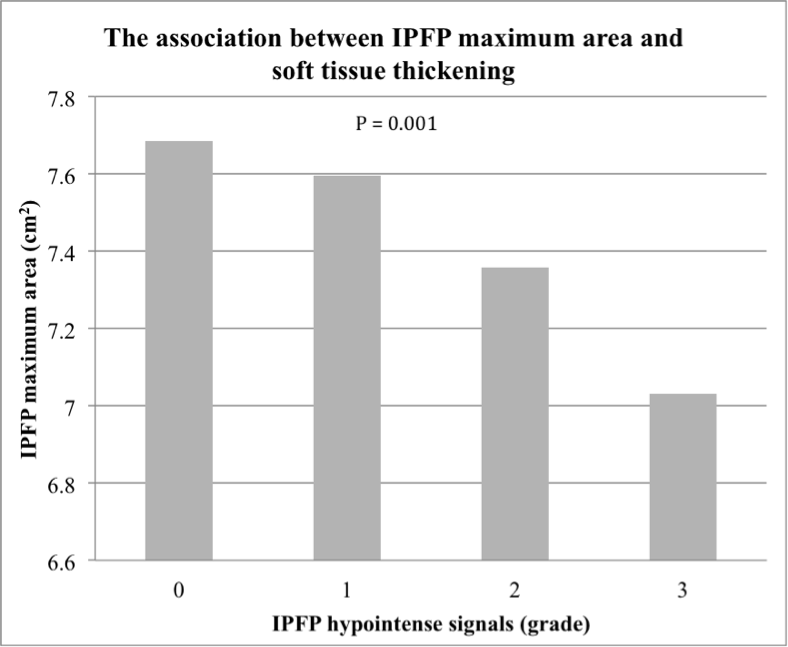

Supplement: Additional file 2: Figure S1. — Association between IPFP maximum area and hypointense signals. IPFP infrapatellar fat pad. (PNG 59 kb) [file 13075_2016_1130_MOESM2_ESM.png]
